# Hematopoietic Stem and Progenitor Cell Expansion in Contact with Mesenchymal Stromal Cells in a Hanging Drop Model Uncovers Disadvantages of 3D Culture

**DOI:** 10.1155/2016/4148093

**Published:** 2015-12-29

**Authors:** Olga Schmal, Jan Seifert, Tilman E. Schäffer, Christina B. Walter, Wilhelm K. Aicher, Gerd Klein

**Affiliations:** ^1^Center for Medical Research, Department of Medicine II, University of Tübingen, 72072 Tübingen, Germany; ^2^Institute of Applied Physics, University of Tübingen, 72076 Tübingen, Germany; ^3^Department of Obstetrics and Gynecology, University of Tübingen, 72076 Tübingen, Germany; ^4^Department of Urology, University of Tübingen, 72076 Tübingen, Germany

## Abstract

Efficient *ex vivo* expansion of hematopoietic stem cells with a concomitant preservation of stemness and self-renewal potential is still an unresolved ambition. Increased numbers of methods approaching this issue using three-dimensional (3D) cultures were reported. Here, we describe a simplified 3D hanging drop model for the coculture of cord blood-derived CD34^+^ hematopoietic stem and progenitor cells (HSPCs) with bone marrow-derived mesenchymal stromal cells (MSCs). When seeded as a mixed cell suspension, MSCs segregated into tight spheroids. Despite the high expression of niche-specific extracellular matrix components by spheroid-forming MSCs, HSPCs did not migrate into the spheroids in the initial phase of coculture, indicating strong homotypic interactions of MSCs. After one week, however, HSPC attachment increased considerably, leading to spheroid collapse as demonstrated by electron microscopy and immunofluorescence staining. In terms of HSPC proliferation, the conventional 2D coculture system was superior to the hanging drop model. Furthermore, expansion of primitive hematopoietic progenitors was more favored in 2D than in 3D, as analyzed in colony-forming assays. Conclusively, our data demonstrate that MSCs, when arranged with a spread (monolayer) shape, exhibit better HSPC supportive qualities than spheroid-forming MSCs. Therefore, 3D systems are not necessarily superior to traditional 2D culture in this regard.

## 1. Introduction

Hematopoietic stem cell (HSC) transplantation is a common treatment procedure for patients suffering from hematopoietic disorders or blood cell cancer [1]. Hematopoietic stem and progenitor cells (HSPCs) derived from umbilical cord blood (UCB) proved to be an effective source for transplantation, combined with the benefit of a minimally invasive recovery method and the possibility of UCB cryopreservation [2–4]. But the small number of available donor cells is often the limiting factor for treatment outcome. Hence, for an efficient* ex vivo* expansion of HSPCs an effective culture method is required which ensures the maintenance of their stemness including the high self-renewal potential.

Hematopoiesis takes place in multiple anatomical regions during embryogenesis. Primitive blood formation starts in the yolk sac and moves to the aorta-gonad-mesonephros region, and definitive hematopoiesis first occurs in the fetal liver [5–7]. During the last trimester of pregnancy, HSPCs migrate from the fetal liver to the circulating blood as hematopoiesis shifts to the bone marrow postnatally. This phenomenon enables the isolation of increased numbers of CD34^+^ HSPCs from UCB.

Endosteal and vascular niches are unique microenvironments in the adult bone marrow that ensure lifelong maintenance and regulation of HSCs through a specialized combination of cellular and molecular components [8, 9]. Bone-forming osteoblasts, bone-resorbing osteoclasts, pericytes surrounding endothelial cells, and mesenchymal stromal cells (MSCs) create a particular extracellular matrix (ECM) and express a variety of cytokines, chemokines, and adhesion receptors regulating HSC quiescence, self-renewal, and differentiation [10–14]. Early long-term culture experiments showed that marrow stromal cells are able to maintain HSC self-renewal and proliferation* in vitro* [15, 16]. More recent studies identified MSCs as key players in the niche in view of the growing number of MSC subpopulations detected in the bone marrow based on their individual expression pattern of CD146, CD140a, CD51, leptin receptor, or nestin [11, 13, 17, 18]. These subpopulations show high potential for HSC maintenance, an ability that designates MSCs as the most frequently used cell type for supporting HSC expansion* ex vivo*.

There are increasing efforts to switch from two-dimensional (2D) to three-dimensional (3D) systems because 3D culture conditions are thought to reflect the* in vivo* situation more accurately, compared with the culture of cells as monolayers. A large diversity of approaches has been reported which have attempted to mimic the inherent HSC environment in a 3D manner via cell encapsulation with hydrogels of natural or artificial origin or self-assembling peptides and polyacrylates [19–22]. Culture devices with low adhesion potential and microwell arrays were tested as 3D models, but some of these should be considered as “quasi-3D models” only [23–25]. Biocompatible macroporous scaffolds which resemble the physiological architecture of trabecular bone seem to more closely represent the natural stem cell habitats [26–28]. However, many of these culture methods are afflicted with disadvantages due to the requirement for complex surface modifications, the use of components of animal origin, or technically demanding and time consuming production processes, making their establishment in routine stem cell laboratories nearly impossible.

In the present study, we sought to evolve an easy-to-use 3D model for the expansion of cord blood-derived HSPCs in coculture with bone marrow-derived MSCs, two cell types which are easily available to most clinical laboratories. Here, we describe a procedure of hanging drop cultures that leads to compact spheroid formation. Cell-cell interactions in the spheroids were visualized by electron microscopy, and synthesis of niche-specific ECM substrates was analyzed using immunofluorescence staining. HSPC proliferation in hanging drops was compared to the coculture in 2D plastic dishes. Finally, colony-forming assays were performed in order to investigate the differentiation potential of HSPCs expanded in the 3D model.

## 2. Materials and Methods

### 2.1. Human Primary Cells and Cell Culture

Umbilical cord blood and bone marrow aspirates were obtained from healthy donors with written informed consent from the Department of Gynecology and Obstetrics or from the BG Trauma Clinic, University of Tübingen, respectively, in accordance with the guidelines of the local ethics committee (reference numbers 005/2012BO2 and 453/2011/BO). Mononuclear cells (MNCs) from cord blood were isolated by Histopaque (1.077 g/mL) density-gradient centrifugation (Sigma-Aldrich, Taufkirchen, Germany) and washed with Dulbecco's phosphate-buffered saline (DPBS; Invitrogen, Life Technologies, Darmstadt, Germany) supplemented with 2 mM EDTA (Biochrom, Berlin, Germany). The MNC population was labeled with anti-CD34-conjugated microbeads according to the instructions of the manufacturer (Miltenyi Biotec, Bergisch Gladbach, Germany). CD34^+^ HSPCs were enriched by magnetic cell separation using MACS columns (Miltenyi Biotec) and used immediately for coculture experiments. MNCs from bone marrow aspirates, enriched by Histopaque (1.077 g/mL) density-gradient centrifugation, were seeded in T75 cell culture flasks in MSC expansion medium compliant with the current good medical procedure regulations (GMP). The GMP medium consisted of DMEM low glucose (Lonza, Basel, Switzerland) supplemented with 5% fresh frozen plasma (TCS Bioscience, Buckingham, United Kingdom), 5% human thrombocyte lysate (Blood Cell Donation Center, University of Tübingen), 2 mM L-glutamine (Lonza), 1000 IE heparin sodium salt (Roth, Karlsruhe, Germany), and 25 mM HEPES sodium salt solution (Sigma-Aldrich). Nonattached cells were removed after 24 hours. Adherent cells were routinely characterized according to the minimum criteria for multipotent MSCs recommended by a consensus conference of the International Society for Cellular Therapy [29]. In the present study, MSCs of passage 2 to passage 4 were used.

### 2.2. 3D Hanging Drop Cultures

After detachment from culture flasks, 5 × 10^3^ MSCs were seeded in 40 *μ*L medium per well of a Perfecta3D 96-well hanging drop plate (3D Biomatrix, Biotrend, Cologne, Germany). The developed spheroids were harvested and analyzed at different time points.

Coculture experiments were performed with a mixture of 5 × 10^3^ MSCs and 5 × 10^2^ CD34^+^ HSPCs per well in 40 *μ*L medium consisting of GMP and serum-free expansion medium (SFEM; Stem Cell Technologies, Grenoble, France) at a 1 : 4 ratio supplemented with the recombinant human cytokines Flt-3 ligand, stem cell factor (100 ng/mL each), interleukin-3, and interleukin-6 (20 ng/mL each) (CC100; Stem Cell Technologies), from here on referred to as “GMP-SFEM-CC100 medium.” Cells were kept in a fully humidified atmosphere with 5% CO_2_ at 37°C. A partial medium exchange was performed every 2 to 3 days.

### 2.3. Inhibition of Spheroid Formation

In a hanging drop plate, 5 × 10^3^ MSCs suspended in 40 *μ*L GMP medium were seeded per well. Cells were treated overnight with the function-blocking monoclonal antibody against the extracellular domain of N-cadherin (clone 8C11; BioLegend, London, United Kingdom) or with the monoclonal anti-cadherin-11 antibody (clone 283416; R&D Systems, Wiesbaden, Germany). Spheroid formation was examined under a light microscope and representative pictures were taken.

### 2.4. Spheroid Harvesting and Cryosectioning

Cryosections of spheroids were used for immunostaining. MSCs alone or in coculture with CD34^+^ HSPCs were incubated in hanging drop plates for up to two weeks. Pictures of MSC spheroids were taken at regular intervals and spheroid diameters were determined using the AxioVision Rel. 4.8 software from Zeiss (Göttingen, Germany). On different days of co- or monoculture, spheroids were washed with PBS supplemented with Ca^2+^ and Mg^2+^ (PBS^++^) in hanging drop plates and fixed with 4% paraformaldehyde (PFA; Electron Microscopy Sciences, Hatfield, USA) for 30 min at room temperature in the dark and stained with Trypan blue (0.5% solution; PAA Laboratories, Cölbe, Germany) for 3 to 5 min, embedded in Tissue-Tek O.C.T. compound (Sakura Finetek Europe, Staufen, Germany), and frozen at –20°C. Cryosections of 5 or 8 *μ*m thickness were air dried for 1 h and stored at –20°C.

### 2.5. Immunofluorescence Staining

Spheroid cryosections were thawed and fixed with 4% PFA for 15 min at room temperature. Samples were incubated for 1 h with primary antibodies diluted in PBS^++^ containing 0.1% bovine serum albumin (BSA; Sigma-Aldrich). In this study, we used the mouse anti-N-cadherin antibody (clone 8C11), the mouse anti-cadherin-11 antibody (clone 283416), and the mouse anti-CD45 (clone HI30; BioLegend) and mouse anti-CD90 (clone 5E10; BioLegend) antibodies. Different laminin chains were detected with the polyclonal rabbit anti-alpha2 chain antiserum (Bioss, Freiburg, Germany) and the mouse anti-alpha4 (clone 3H2) and anti-alpha5 chain (clone 4B12) antibodies (both kindly provided by Dr. Sulev Ingerpuu, IMCB, University of Tartu, Estonia). ECM components were stained with the rabbit anti-collagen type IV (kind gift of Dr. Johannes Eble, University of Münster, Germany) and rabbit anti-collagen type VI [30] antibodies. The mouse anti-fibronectin (clone P1H11) and the mouse anti-tenascin-C (clone T2H5) antibodies were obtained from R&D Systems and Abcam (Cambridge, United Kingdom), respectively. For apoptosis analysis, sections were permeabilized using 0.1% Triton X-100 (AppliChem; Schubert & Weiss, Munich, Germany), incubated for 1 h in blocking buffer (5% normal goat serum, 0.3% Triton X-100 in PBS), and stained overnight with the rabbit antibody against cleaved caspase-3 (clone 5A1E) or the antibody detecting cleaved PARP (clone D64E10; both from Cell Signaling Technology; New England Biolabs, Frankfurt, Germany) at +4°C. After washing with PBS^++^, bound primary antibodies were detected by Cy3-conjugated goat anti-mouse, Cy3-conjugated goat anti-rabbit, and Alexa Fluor 488-conjugated goat anti-rabbit antibodies (Jackson ImmunoResearch; Dianova, Hamburg, Germany). Cell nuclei were identified by counterstaining with 4′,6-diamino-2-phenylindole-dihydrochloride (DAPI, 1 *μ*g/mL; Roche, Mannheim, Germany). Primary antibodies were omitted for control staining. Photographs were taken using the Axiophot microscope (Zeiss).

### 2.6. Scanning Electron Microscopy (SEM)

CD34^+^ HSPCs were incubated with MSCs in hanging drop plates at a ratio of 0.5 : 5 × 10^3^ for up to 7 days. At different time points, coculture samples were prepared for SEM analysis. Cells in hanging drops were washed with PBS^++^, fixed with Karnovsky's fixative (2% paraformaldehyde (Electron Microscopy Sciences), 2.5% glutaraldehyde (Serva, Heidelberg, Germany)) for 30 min at room temperature in the dark, washed with PBS^++^, and stored in 70% ethanol at +4°C until all samples were collected. Dehydration was performed with an increasing graded ethanol series. The samples were dried at room temperature, immobilized to coverslips via a one-component cyanoacrylate adhesive (Wevo-Cyamet 75; Wevo-Chemie, Ostfildern, Germany), and mounted onto aluminum holders using conductive tabs (G3347, Plano, Wetzlar, Germany) and then sputter coated with a 20 nm gold layer. Analysis was performed using a scanning electron microscope (XL30, Philips, Amsterdam, Netherlands). SEM images were recorded with an acceleration voltage of 10 kV.

### 2.7. MSC Proliferation Analysis

5 × 10^3^ MSCs suspended in 40 *μ*L GMP-SFEM-CC100 medium were seeded in parallel in a hanging drop plate and in a conventional 96-well flat-bottom plate and cultivated in a humidified environment with 5% CO_2_ at 37°C. Partial medium changes were performed as required. On day 3 and day 7, spheroids were harvested by pipetting into a 96-well plate. Medium was removed from the 2D and 3D cultures and plates were directly transferred to –80°C. The MSC proliferation rate was determined by analysis of DNA content (performed in triplicate) using the CyQUANT kit from Invitrogen following the manufacturer's instructions. A calibration curve was generated by seeding different MSC numbers in a 96-well plate.

### 2.8. HSPC Expansion Analysis

5 × 10^2^ CD34^+^ HSPCs were seeded in 40 *μ*L GMP-SFEM-CC100 medium per well either alone (monoculture) or together with 5 × 10^3^ MSCs (coculture) in a flat-bottom or a hanging drop 96-well plate (2D or 3D, resp.). On days 4, 7, 10, and 14, the HSPC proliferation rate was determined by manually counting the number of cells under a light microscope (performed in triplicate). Cell aggregations in the 3D culture were separated by pipette mixing, and cells in the 2D culture were detached by incubation with Accutase solution (Sigma-Aldrich) for 3 to 5 min. HSPCs were clearly distinguishable from MSCs based on cell size, shape, and granularity.

### 2.9. Colony-Forming Assay

In order to analyze the influence of 2D and 3D culture conditions on the differentiation potential of hematopoietic progenitors, colony-forming assays were performed with HSPCs expanded in triplicate in coculture with MSCs for one week. 10^3^ HSPCs per replicate were diluted in 100 *μ*L Iscove's modified Dulbecco's medium plus 2% fetal bovine serum (Stem Cell Technologies) and added to 1 mL methylcellulose medium containing recombinant human stem cell factor, granulocyte-macrophage colony-stimulating factor, interleukin-3, and erythropoietin (MethoCult H4434, Stem Cell Technologies). Cells plated in 35 mm petri dishes were cultured in a fully humidified environment with 5% CO_2_ at 37°C for 14 days. Cell aggregates containing more than 50 cells were identified as single colonies using an inverted microscope (Axiovert 135; Zeiss). Burst-forming unit erythrocyte (BFU-E) and colony-forming unit-granulocyte/macrophage (CFU-GM) colonies as well as colonies arising from multipotent granulocyte, erythrocyte, macrophage, and megakaryocyte progenitors (CFU-GEMM) were counted independently by two investigators on the basis of morphological criteria. The differentiation potential of cells expanded in 2D or 3D was compared to freshly isolated, nonexpanded CD34^+^ HSPCs. The CFU fold increase for the different progenitors was calculated as follows:

(1) $$\begin{array}{c} \frac{\text{CFU number of expanded HSPCs}\times \text{proliferation factor in 2D or 3D after one week}}{\text{CFU number of nonexpanded HSPCs}}=\text{CFU fold increase}. \end{array}$$

### 2.10. Immunoblotting

Total protein extracts were obtained by incubating confluent MSC cultures with extraction buffer containing 1% NP-40, 150 mM NaCl, 40 mM Tris, 2 mM EDTA, 0.5% sodium deoxycholate, 0.1% SDS (pH 8), and a proteinase inhibitor cocktail (Roche). Cell lysates were diluted in a DTT-containing sample buffer and run on 6% SDS polyacrylamide gels. PVDF membranes (Merck Millipore, Schwalbach, Germany) were used for blotting. Unspecific binding sites were blocked with Tris-buffered saline containing 0.1% Tween-20 and 5% skimmed milk powder (Roth). Blot membranes were incubated with the mouse antibodies against N-cadherin and cadherin-11 overnight at +4°C. Bound primary antibodies were detected with HRP-conjugated or AP-conjugated anti-mouse antibodies (Dako, Hamburg, Germany) and the chemiluminescence reagent Immobilon Western (Merck Millipore) or the BCIP/NBT substrate (Sigma), respectively.

### 2.11. Statistical Analysis

Values are expressed as mean ± standard deviation (SD). Statistical significance was determined by two-tailed parametric *t*-tests or one-way ANOVA using GraphPad Prism 5 software (Version 5.01). Differences were considered to be significant for ^*∗*^ *p* < 0.05, ^*∗∗*^ *p* < 0.01, and ^*∗∗∗*^ *p* < 0.001 with increasing degrees of significance.

## 3. Results

### 3.1. Hanging Drop Culture of MSCs Leads to Spheroid Formation and Proliferation Arrest

**Figure 1:**
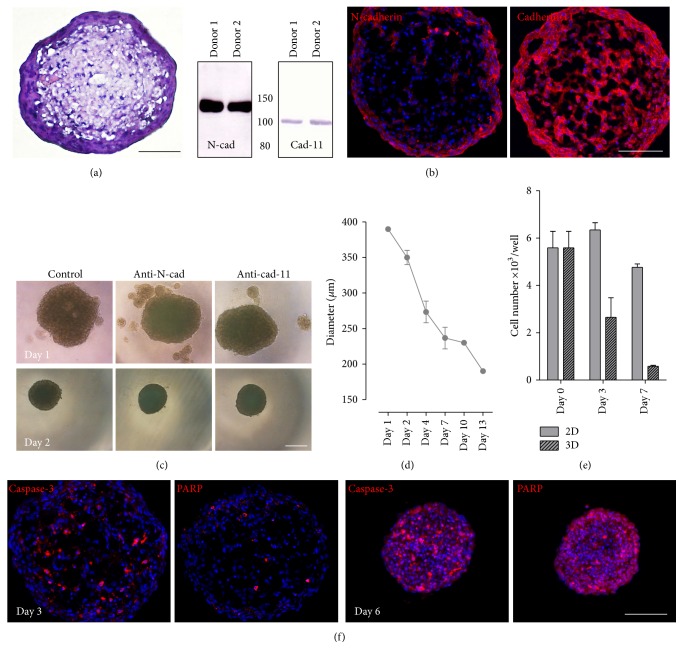
MSCs cultured in hanging drop plates form stable spheroids and do not proliferate. 5 × 10^3^ mesenchymal stromal cells seeded in a hanging drop plate aggregate into a spheroid. (a) Hematoxylin/eosin staining of a spheroid cryosection shows a typical morphology with vast intercellular spaces in the central region and a tight peripheral MSC ring after 3 days of culture. (b) Human bone marrow MSCs express the cell adhesion molecules N-cadherin and cadherin-11 as shown by immunoblotting of lysates from confluent cell layers of two different donors. Using immunofluorescence staining, both cadherins were clearly detectable in MSC spheroids. (c) Aggregation of MSCs into spheroids was investigated in the presence of antibodies against N-cadherin and cadherin-11. On day 1, additional smaller aggregates were detectable as compared to the untreated cells, while all spheroids showed a similar morphology on the second culture day. (d) Determination of the diameter of the formed MSC spheroids with the AxioVision software revealed a continual decrease over two weeks. Spheroid sizes of four different donors were analyzed. Data are means ± SD. (e) Cell numbers of MSCs cultured in GMP-SFEM-CC100 medium as hanging drops (3D) or in conventional 2D plates were quantified in triplicate and are shown as means ± SD. The data are representative of three donors with comparable results. (f) Apoptotic cells in 3- and 6-day-old spheroids were detected by staining with specific anti-caspase-3 and anti-PARP antibodies. Cell nuclei were counterstained with DAPI. The immunofluorescence and light microscopy pictures are representative for MSC spheroids of at least three different donors. Scale bars: 100 *μ*m (a, b, and f) and 250 *μ*m (c).

Prior to the 3D coculture, the behavior of MSCs in the hanging drop model was examined. 5 × 10^3^ cells were seeded in 40 *μ*L medium per well of a 96-well hanging drop plate. Within one day, the MSCs readily aggregated into a single compact spheroid of approximately 380–400 *μ*m in diameter. Hematoxylin/eosin staining of cryosections revealed a spongy core of the spheroid with bulky intercellular spaces surrounded by a tight ring of MSCs (Figure 1(a)). The cell adhesion molecules N-cadherin and cadherin-11, earlier shown to mediate the interaction of human HPCs and MSCs [31], were both expressed by bone marrow-derived MSCs as confirmed by Western blotting (Figure 1(b)). Immunofluorescence staining of cryosections showed an even distribution of cadherin-11 throughout the spheroid, whereas the N-cadherin signal was more prominent in the periphery and rather faint in the spongy core (Figure 1(b)). Nevertheless, MSC spheroid formation was impaired to a higher extent by a function-blocking anti-N-cadherin antibody than after addition of an anti-cadherin-11 antibody, leading to the formation of additional small aggregates (Figure 1(c)). This inhibitory effect, however, was short-lived because no differences were detectable on day 2 compared to the untreated spheroids. These findings show that MSCs devoid of any substrate give rise to compact aggregates with tight homotypic interactions. N-cadherin and cadherin-11 are at least partly responsible for the observed cell-cell contact formation, but it is likely that additional cell adhesion molecules are also involved.

After spheroid formation was completed, the diameter of the cell aggregate decreased gradually during the following two weeks of culture to almost half of the original size (Figure 1(d)), indicating that, in contrast to their high expansion rate under 2D culture conditions, MSCs do not proliferate in this 3D model. Indeed, quantification of DNA content after 3 and 7 days revealed considerably lower cell numbers per spheroid compared with the MSC numbers in the starting culture (Figure 1(e)). This assay was performed in GMP-SFEM-CC100 medium at a 1 : 4 ratio because this mixture was identified as the optimum for HSPC expansion in the coculture studies (Supplementary Figure S3 (see Supplementary Material available online at http://dx.doi.org/10.1155/2016/4148093)). The MSC growth cessation in spheroids was only partly due to the medium composition and was mainly a consequence of the 3D culture conditions because spheroids incubated in the MSC expansion medium GMP exhibited the same growth behaviour (Supplementary Figure S1). The proliferation arrest in spheroids was accompanied by an onset of apoptotic events. The nuclear protein PARP1 can be processed by proteolytic activation of caspase-3, a key step during apoptosis induction. Caspase-3 and cleaved PARP1-positive cells could be detected in the central region of 3-day-old spheroids (Figure 1(f)). The number of apoptotic cells was clearly increased after one week. In summary, MSC spheroids were characterized by a continually shrinking diameter due to aggregate compaction (comparing days 3 and 6 in Figure 1(f)), lack of proliferation, and the onset of apoptosis.

### 3.2. Cord Blood HSPCs Disrupt MSC Spheroids in the Hanging Drop Coculture

**Figure 2:**
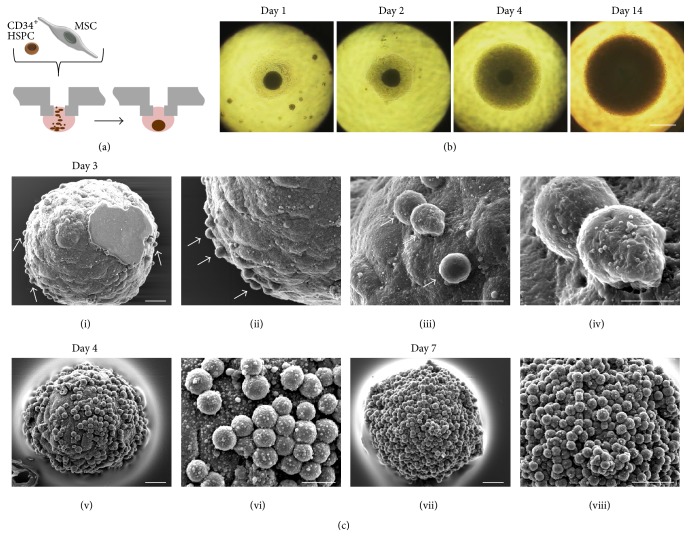
Attachment of CD34^+^ HSPCs to newly formed spheroids increases with time. (a) For coculture experiments, 5 × 10^2^ CD34^+^ HSPCs were seeded together with 5 × 10^3^ bone marrow MSCs per hanging drop and incubated for up to two weeks in GMP-SFEM-CC100 medium. (b) Light microscopy images depict the aggregation of MSCs into spheroids in the coculture as soon as one day after culture. Surrounding HSPCs expanded greatly over time. After 14 days, the cell density in the hanging drop was very high and the spheroid in the center became invisible. Scale bar: 500 *μ*m. (c) Spheroids formed in cocultures were examined by scanning electron microscopy (SEM). A series of images from day 3 with increasing magnification (i–iv) shows a spheroid with only a few adhering HSPCs, identified as small round cells (arrows) attaching to the surface of the relatively smooth spheroid. On day 4, significantly more HSPCs attached to the spheroid (v, vi). After 7 days, the whole surface of the spheroid was covered by HSPCs, which also seem to attach to each other (vii, viii). Scale bars: 25 *μ*m (i, ii, v, vii, and viii); 10 *μ*m (iii, vi); and 5 *μ*m (iv).

CD34^+^ HSPCs isolated from umbilical cord blood were seeded together with bone marrow-derived MSCs at a ratio of 5 × 10^2^ : 5 × 10^3^ per well in a 96-well hanging drop plate and cultured in GMP-SFEM-CC100 for 2 weeks (Figure 2(a)). As described for MSCs alone, the MSCs in the coculture similarly aggregated into compact spheroids which were surrounded by rapidly expanding HSPCs (Figure 2(b)). Scanning electron microscopy (SEM) revealed that, at the beginning (day 3), MSC spheroids exhibited a smooth surface with only a few HSPCs attached to it (Figure 2(c)). With increasing time, HSPC adhesion increased (day 4) until after one week of coculture when the entire spheroid surface was covered with HSPCs. Here, the hematopoietic cells attached not only to the MSCs but also to one another (Figure 2(c)).

**Figure 3:**
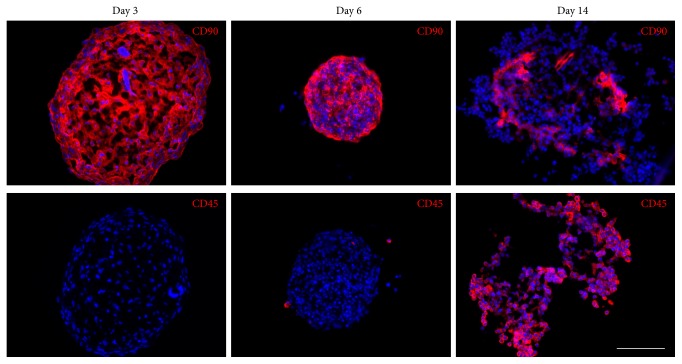
HSPCs, initially excluded by spheroid-forming MSCs, penetrate the spheroids after one week of coculture. Immunofluorescence staining of spheroid cryosections with antibodies against CD90 detecting MSCs and CD45 detecting hematopoietic cells revealed that on day 3 of coculture the spheroids were exclusively made up of MSCs. After 6 days of coculture, compaction of the spheroid occurred and CD90 staining was still present throughout the spheroid. Only a few CD45^+^ cells were attached to the spheroid surface. On day 14 of coculture, most cells were stained with the anti-CD45 antibody, while the CD90^+^ cells of the core seemed to be collapsed. Cell nuclei were counterstained with DAPI. Scale bar: 100 *μ*m.

For the analysis of spheroid composition and for determination of the localization of both cell types inside of the aggregate, cryosections were immunostained with antibodies against CD90, a MSC-specific surface marker, and against CD45, a pan-hematopoietic marker, for which MSCs are known to be negative. During the initial phase of coculture, there was no evidence of HSPC occurrence inside MSC spheroids because no CD45 signal was detectable at day 3 (Figure 3). Instead, all cells were positive for CD90. The spheroid morphology already described in the previous section was evident with a dense peripheral MSC ring which presumably prevented HSPC invasion. This morphology turned steadily into a more compact MSC aggregation resulting in smaller spheroid diameters. On day 6, CD45^+^ HSPCs occurred at the spheroid surface (Figure 3). After two weeks of coincubation, the vast majority of cells in the aggregate were CD45^+^, whereas CD90 showed a faint patchy staining intensity, indicating a collapse of the MSC core (Figure 3). In conclusion, the HSPC adhesion strength in this 3D hanging drop model is rather weak because they could easily be dissociated from the MSC spheroid by extensive pipetting and without the addition of digesting enzymes. In spite of a mixed MSC-HSPC suspension at the seeding time point, the two cell types segregated and rearranged reproducibly, indicating that the homotypic interactions of MSCs outweigh their contact to hematopoietic cells.

### 3.3. MSC Spheroids Synthesize Niche-Specific ECM

**Figure 4:**
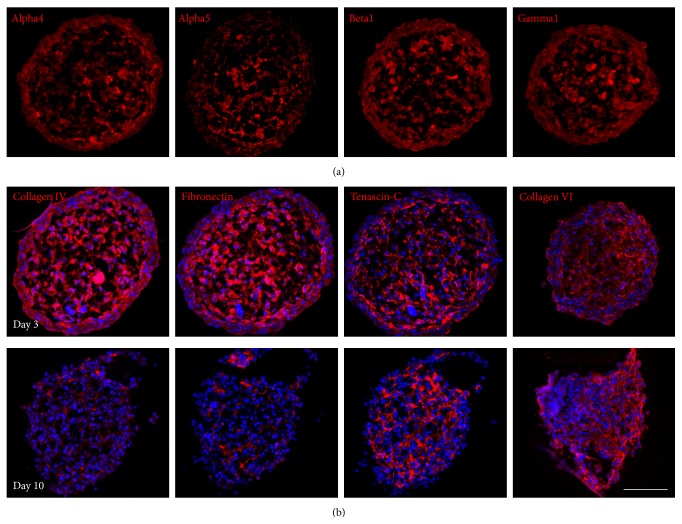
MSC spheroids express ECM components of the bone marrow. (a) The micrographs show immunofluorescence staining of 3-day-old MSC spheroid cryosections labeled with laminin chain-specific antibodies. Strong signals for the laminin alpha4, alpha5, beta1, and gamma1 chain were detected throughout the spheroids. (b) Sections from coculture spheroids harvested on days 3 and 10 were stained with antibodies against collagen type IV, fibronectin, tenascin-C, and collagen type VI. While all four matrix components were strongly expressed in early spheroids (upper panel), prominent expression was only detected for tenascin-C and collagen type VI in later stages of coculture (lower panel). Cell nuclei were counterstained with DAPI. Scale bar: 100 *μ*m.

The expression pattern of bone marrow ECM components inside the spheroids was investigated by immunofluorescence staining. In adult human bone marrow, the laminin isoform LM511 was identified as an essential constituent of the ECM [32, 33]. Immunofluorescence staining of spheroid cryosections with laminin chain-specific antibodies revealed strong signals for the alpha5, beta1, and gamma1 chains (Figure 4(a)). The laminin alpha4 chain, also expressed by bone marrow cells [33], has been implicated in adhesion and migration of hematopoietic progenitors [32]. The laminin alpha4 chain was observed to be present to a high degree in MSC spheroids (Figure 4(a)). The laminin alpha2, beta2, and gamma2 chains, also described in the bone marrow [34, 35], were absent in the spheroids (Supplementary Figure S2). In summary, MSCs cultured as spheroids vigorously express LM411 and LM511.

The basement membrane component collagen type IV was detectable throughout the early MSC spheroid and was expressed to a similarly high degree as fibronectin, a basic constituent of the bone marrow extracellular matrix (Figure 4(b)). In addition, 3-day-old spheroids synthesized considerable amounts of the large glycoprotein tenascin-C and the microfibrillar collagen type VI. Both ECM proteins display strong cytoadhesive properties for hematopoietic progenitor cells [30, 36]. MSC spheroids were also positive for the fibrillar collagen type I and for the proteoglycan perlecan (Supplementary Figure S2B), an integral part of basement membranes, which is an antiadhesive bone marrow substrate [37]. After 10 days in culture, expression of most ECM molecules was clearly reduced except for tenascin-C und collagen type VI (Figure 4(b)). Both components still produced a scaffold-like structure, thus probably allowing HSPC aggregation even after breakdown of the MSC core. These results demonstrate that various principal bone marrow ECM constituents involved in HSPC adhesion and regulation are synthesized by early MSC spheroids during hanging drop culture.

### 3.4. HSPC Expansion in 2D Coculture Exceeds the Proliferation Rate in Hanging Drops

**Figure 5:**
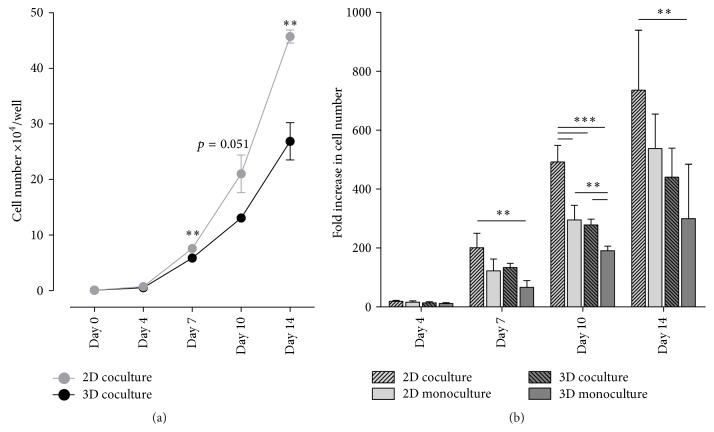
HSPCs proliferate more extensively in coculture with MSC monolayers than with MSC spheroids. Expansion of HSPCs under 2D or 3D conditions was compared by seeding 500 HSPCs in combination with 5000 MSCs in conventional 96-well culture plates or in hanging drop plates, respectively, followed by determination of HSPC numbers at different time points. (a) Representative data from one donor show a significantly lower proliferation rate already after one week of coculture in 3D when compared with 2D. Samples were analyzed in triplicate. Statistical analysis was performed using a two-tailed parametric *t*-test. (b) The fold increase in HSPC numbers was obtained after expansion of 500 HSPCs in coculture or in monoculture. The highest proliferation rates were detected for the 2D coculture, whereas HSPCs incubated with MSC spheroids in 3D did not exceed the expansion level of the 2D monoculture. Statistical significance was assessed using one-way ANOVA. Data are shown as mean ± standard deviation of four independent experiments with four donors (^*∗∗*^ *p* < 0.01; ^*∗∗∗*^ *p* < 0.001).

A primary goal of the 3D coculture model was fast and efficient expansion of cord blood-derived CD34^+^ HSPCs. In initial studies, a mixture of one volume MSC expansion medium GMP with four volumes HSPC expansion medium SFEM, supplemented with 100 ng/mL Flt-3L and SCF, and 20 ng/mL IL-3 and IL-6 (GMP-SFEM-CC100) yielded the highest proliferation rate (Supplementary Figure S3B). For comparison with 2D cultures, the MSC-HSPC suspension was seeded in the same volume with the same cell numbers in a conventional flat-bottom 96-well plate, in which MSCs readily adhered to the well bottom and HSPCs evenly attached to the MSC monolayer (Supplementary Figure S3C). After one week of coincubation, numbers of expanded HSPCs per hanging drop were already significantly lower than in a comparable 2D well (Figure 5(a)). This difference became even more prominent after 14 days of culture ((26.9 ± 3.4) × 10^4^ cells under 3D and (45.7 ± 1.2) × 10^4^ cells under 2D conditions, resp.). Coculture of HSPCs with MSCs was beneficial compared with HSPCs as a monoculture in the corresponding system (Figure 5(b)). The positive effect on proliferation was more prominent when CD34^+^ HSPCs were coincubated with a MSC monolayer (736 ± 204 fold increase) than with a MSC spheroid (440 ± 98 fold increase). In the 2D monoculture system, the expanding HSPCs accumulate in one half of the well whereas in the 2D coculture an even HSPC distribution over the entire well area could be observed (Supplementary Figure S3C) indicating that direct cell-cell contact between HSPCs and MSCs was essential for an effective proliferation of the former.

### 3.5. 3D Coculture Does Not Favor Expansion of Primitive Progenitors

**Figure 6:**
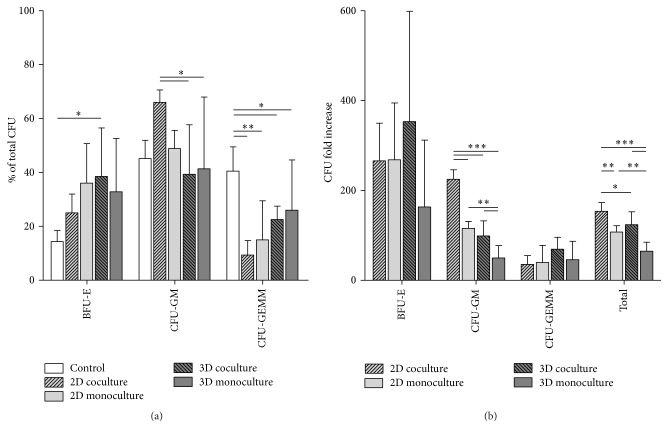
The 3D model is not favorable for the enhancement of primitive progenitors. Multilineage differentiation capacity of 10^3^ HSPCs expanded in the hanging drop model or in a 2D culture plate was investigated after one week of culture with or without MSCs (coculture and monoculture, resp.) in a CFU assay. Results were compared to the CFU forming potential of 10^3^ freshly isolated cord blood-derived HSPCs (control). (a) The BFU-E percentage of the total CFU number increased after* ex vivo* expansion at the expense of CFU-GEMM for all culture conditions. The CFU-GM percentage of the total CFU number was considerably augmented only for HSPCs in 2D coculture. (b) The CFU fold increase was obtained by comparison with colony numbers of nonexpanded cells and was calculated using the proliferation rate of HSPCs under the respective culture conditions. HSPCs expanded on an adherent MSC layer produced the largest number of total colonies. Values are expressed as mean ± standard deviation of three independent experiments from three donors. One-way ANOVA was applied for statistical analysis (^*∗*^ *p* < 0.05; ^*∗∗*^ *p* < 0.01; ^*∗∗∗*^ *p* < 0.001).

Multilineage differentiation potential of expanded HSPCs can be analyzed by colony-forming unit (CFU) assays. After 14 days of incubation, colonies deriving from erythroid progenitors (BFU-E), granulocyte/macrophage progenitors (CFU-GM), or multilineage granulocyte/erythrocyte/macrophage/megakaryocyte progenitors (CFU-GEMM) can be identified based on their morphological appearance and hemoglobin production. CFU assays with HSPCs expanded either as co- or monoculture under 2D and 3D conditions for 7-8 days were performed because differences in proliferation between the respective methods were already significant at this time point.  Figure 6(a) depicts the distribution of the particular CFUs arising from unexpanded cells or from cells expanded in 2D or 3D, expressed as percentage of total colony counts. A general trend was observed for increasing BFU-E after expansion at the expense of CFU-GEMM. This coincides with the high proliferative capacity of erythroid progenitors from cord blood when compared to stem cells originating from other sources [38]. HSPCs expanded on a MSC monolayer produced significantly more CFU-GM than those incubated in hanging drops. When the proliferation rate of HSPCs after one week in the respective system was taken into account and the CFU profile was expressed as a fold increase of colony numbers developed from 10^3^ freshly isolated, unexpanded HSPCs (Figure 6(b)), the difference in CFU-GM was even more pronounced (225 ± 21 fold increase in 2D coculture; 116 ± 15 in 2D monoculture; 99 ± 34 in 3D coculture; 50 ± 27 in 3D monoculture). The fold increase of BFU-E and CFU-GEMM tended to be higher after expansion in the 3D coincubation system but failed to reach statistical significance. In contrast, the total colony number for HSPCs deriving from the 2D coculture was significantly elevated in comparison to all other culture methods. According to these results, HSPC expansion with MSC spheroids in hanging drops has no significantly beneficial effect on their colony-forming potential over cells grown with MSC monolayers.

## 4. Discussion

Fast and efficient* ex vivo* expansion of hematopoietic stem cells prior to transplantation is still a challenge which many research groups have attempted to overcome by placing more and more emphasis on 3D techniques. In the present study, we analyzed a simplified 3D coincubation model of cord blood-derived CD34^+^ HSPCs together with bone marrow-derived MSCs in a hanging drop culture which led to an aggregation of MSCs into spheroids surrounded by HSPCs. Surprisingly, HSPC expansion in a conventional 2D culture system was higher than the proliferation rate in the 3D model, although the expression of many essential bone marrow ECM components was detected in the spheroids. Furthermore, HSPCs expanded in hanging drop plates were inferior to those from 2D culture with respect to their differentiation potential. Accordingly, in contrast to the widely accepted dogma, our study displayed that traditional 2D culture might, in some aspects, be advantageous over certain 3D systems.

The hanging drop culture method has been greatly improved by creating well plates with perforated arrays in which hanging drops are formed by gravity forces. These plates are currently a widely used culture system in tumor biology and drug testing studies and allow the application of automated liquid handling systems for high-throughput analyses [39–41]. Furthermore, this system is advantageous over other spheroid-based methods like low-adhesion culture devices or specific surface coatings due to its outstanding reproducibility by formation of uniquely sized aggregates. To the best of our knowledge, our study is the first that uses the hanging drop method for mimicking the HSC niche* in vitro*.

A recent study reported elevated expression levels of anti-inflammatory and anticancer factors by 3-day-old MSC spheroids [42]. Comparable to our findings, Bartosh and coworkers also observed spheroid compaction and the appearance of an epithelial-like layer on the surface [43]. Enhanced differentiation potential of MSCs into the adipogenic and osteogenic lineages or into epithelial and neuronal progenitors was also demonstrated with the spheroid incubation method [44, 45]. These reports emphasize that when MSCs are grown as monolayers or in 3D systems, changes occur not only in cell morphology and structure but also in transcriptome and proteome profiles and consequently in cell behavior. Cell growth most likely decreases in a 3D environment due to contact inhibition because most cells experience close interactions with several adjacent neighbors, which might explain the absence of MSC proliferation in the hanging drop spheroids. Plastic/substrate adherence is one of the characteristic criteria for highly expanding MSCs. Hence, reduced or a lack of opportunities to firmly attach to a substrate might additionally impair their expansion potential.

The ECM is an essential constituent of the hematopoietic stem cell niches. Therefore the question arose whether exogenous ECM components should be added to the hanging drop culture. Matrigel is a widely used tool to create a 3D environment [46]. But due to its varying compositions of diverse ECM molecules, the use of Matrigel might hinder reproducibility [47]. Our 3D model shows that, within the spheroids, MSCs produce considerable amounts of ECM components also found in the hematopoietic niches* in vivo*. Notably the laminin isoforms LM411 and LM511, which are implicated in HSPC adhesion and migration [32, 33], were already detected in 3-day-old spheroids. Tenascin-C and fibronectin were also strongly expressed in the newly formed spheroids. Adhesion of HSPCs to these matrix components is mediated via the integrins *α*4*β*1 and *α*9*β*1, respectively, which also regulate the proliferation of HSPCs [48, 49]. The bone-specific collagen type I and the microfibrillar collagen type VI, which both show cytoadhesive properties for HSPCs [30, 50], as well as the antiadhesive proteoglycan perlecan [37] were also expressed in the spheroids.

Nevertheless, HSPCs did not migrate into the early spheroids and showed only low adhesive affinity for the MSCs at the beginning of the coculture. In the following time points, HSPC attachment increased considerably, which coincided with distinct spheroid compaction. Therefore, another important factor for HSPCs, substrate elasticity, should be considered in the comparison between 2D and 3D models. HSPCs have been shown to sense the biomechanical properties of their microenvironment and to transduce these signals into cellular responses [51]. Alterations in substrate elasticity can change adhesive interactions and migration of human HSPCs [52]. On the other hand, MSCs also decide upon their cell fate according to matrix elasticity and cell geometry [53, 54] which in turn may influence their HSPC-regulating capabilities. It is tempting to speculate that HSPCs sense a higher stiffness of the compact MSC spheroids and show increased attachment. This would be in line with the observation made in the 2D culture where almost all HSPCs adhered to the MSC monolayer from the beginning of the coculture, because cells spread on plastic are certainly less elastic than cells aggregated into a sphere with large intercellular spaces. Future studies using atomic force microscopy are required to test the correctness of these speculations.

Distinct MSC subpopulations of the human bone marrow were recently described to form differentially sized mesenspheres during* in vitro* culture and to promote HSPC expansion [18, 55]. The proliferation-promoting ability of these aggregates was not dependent on cell-cell contacts. In contrast, our results support the need for a direct MSC-HSPC interaction, in line with reports from other groups [56, 57]. HSPCs expanded to a higher extent when grown on a MSC monolayer, thereby experiencing a larger contact area than their counterparts in coculture with MSCs as a spheroid. Similarly, HSPCs expanded in the 2D coculture contained remarkably more granulocyte/macrophage progenitors and produced significantly higher total colony numbers. The hanging drop coculture was only slightly superior in the content of erythroid and mixed progenitors. To summarize, the respective culture conditions supported distinct hematopoietic progenitors, and direct MSC contact apparently plays a different role in HSPC differentiation than in HSPC proliferation.

For a more authentic reproduction of the niche* in vitro*, supplement of additional bone marrow cells such as endothelial cells may be required. Recently, transplantable units isolated from mouse bone marrow with mesenchymal and hematopoietic stem cell properties were shown to be frequently associated with blood vessels [58]. However, incorporation of additional cell types will unavoidably increase the complexity of every model and hamper its realization due to restricted material availability.

## 5. Conclusions

The presented 3D hanging drop model of MSCs and HSPCs provides evidence that 3D culture is not always superior to 2D conditions. For HSPC expansion especially, coculture with a MSC monolayer was more efficient than with a MSC spheroid. Our findings imply that direct contact of both cell types is required for HSPC expansion. Although the spheroid-forming MSCs are fully capable of synthetizing niche-specific ECM components, their strong homotypic interactions seem to prevent an extensive contact with HSPCs. MSC morphology, expression of surface molecules, and their interaction with HSPCs are different when MSCs are grown as a spread monolayer compared to the case when grown as a spheroid where the cells are of reduced volume and surface area. It appears that MSCs need a physical substrate in order to optimally exert their HSPC supportive functions, an important point that should be considered for* ex vivo* expansion protocols.

## Highlights

- During MSC/HSPC coculture in hanging drops, MSCs segregate into tight spheroids.
- HSPCs are initially excluded from the MSC spheroids but replace MSCs at later stages.
- HSPC expansion in 3D culture is lower than in 2D coculture.
- The largest number of colony-forming units is found after 2D coculture.

## Supplementary Material

Supplementary material contains a graph comparing MSC growth in the expansion medium GMP under 2D and 3D conditions (S1). Spheroid-forming MSCs were characterized by a clear proliferation arrest compared to a monolayer indicating that the observed growth cessa-tion is a consequence of 3D culture conditions and not of medium composition.
Expression of further niche-specific ECM components (laminin chains alpha2, beta2, gamma2; perlecan and collagen type I) in spheroids was investigated by means of immunofluorescence (S2).
Supplementary Figure S3 describes the method applied for the determination of HSPC proliferation rates under 2D and 3D conditions and the evaluation of the proper medium composition yielding the highest proliferation rate in the 3D model. Remarkable differences in HSPC distribution were observed in the 2D system when the cells were grown alone or on a MSC monolayer indicating the importance of direct cell-cell contacts for their proliferation.
