# Supplementary material for: Hematopoietic Stem and Progenitor Cell Expansion in Contact with Mesenchymal Stromal Cells in a Hanging Drop Model Uncovers Disadvantages of 3D Culture
Source: Stem Cells Int. 2015 Dec 29;2016:4148093. doi: 10.1155/2016/4148093 (PMC4709770; doi:10.1155/2016/4148093)
Supplement: Supplementary file 1 — Supplementary material contains a graph comparing MSC growth in the expansion medium GMP under 2D and 3D conditions (S1). Spheroid-forming MSCs were characterized by a clear proliferation arrest compared to a monolayer indicating that the observed growth cessa-tion is a consequence of 3D culture conditions and not of medium composition. Expression of further niche-specific ECM components (laminin chains alpha2, beta2, gamma2; perlecan and collagen type I) in spheroids was investigated by means of immunofluorescence (S2). Supplementary Figure S3 describes the method applied for the determination of HSPC proliferation rates under 2D and 3D conditions and the evaluation of the proper medium composition yielding the highest proliferation rate in the 3D model. Remarkable differences in HSPC distribution were observed in the 2D system when the cells were grown alone or on a MSC monolayer indicating the importance of direct cell-cell contacts for their proliferation. [file 4148093.f1.docx]

**Supplementary material to**

**Hematopoietic stem and progenitor cell expansion in contact with mesenchymal stromal cells in a hanging drop model uncovers**

**disadvantages of 3D culture**

**Olga Schmal,^1^ Jan Seifert,^2^ Tilman E. Schäffer,^2^ Christina B. Walter,^3^**

**Wilhelm K. Aicher,^4^ and Gerd Klein^1^**

^1^ Center for Medical Research, Department of Medicine II, University of Tübingen

^2^ Institute of Applied Physics, University of Tübingen

^3^ Department of Obstetrics and Gynecology, University of Tübingen, Germany

^4^ Department of Urology, University of Tübingen, Germany

**Supplementary figures and figure legends:**


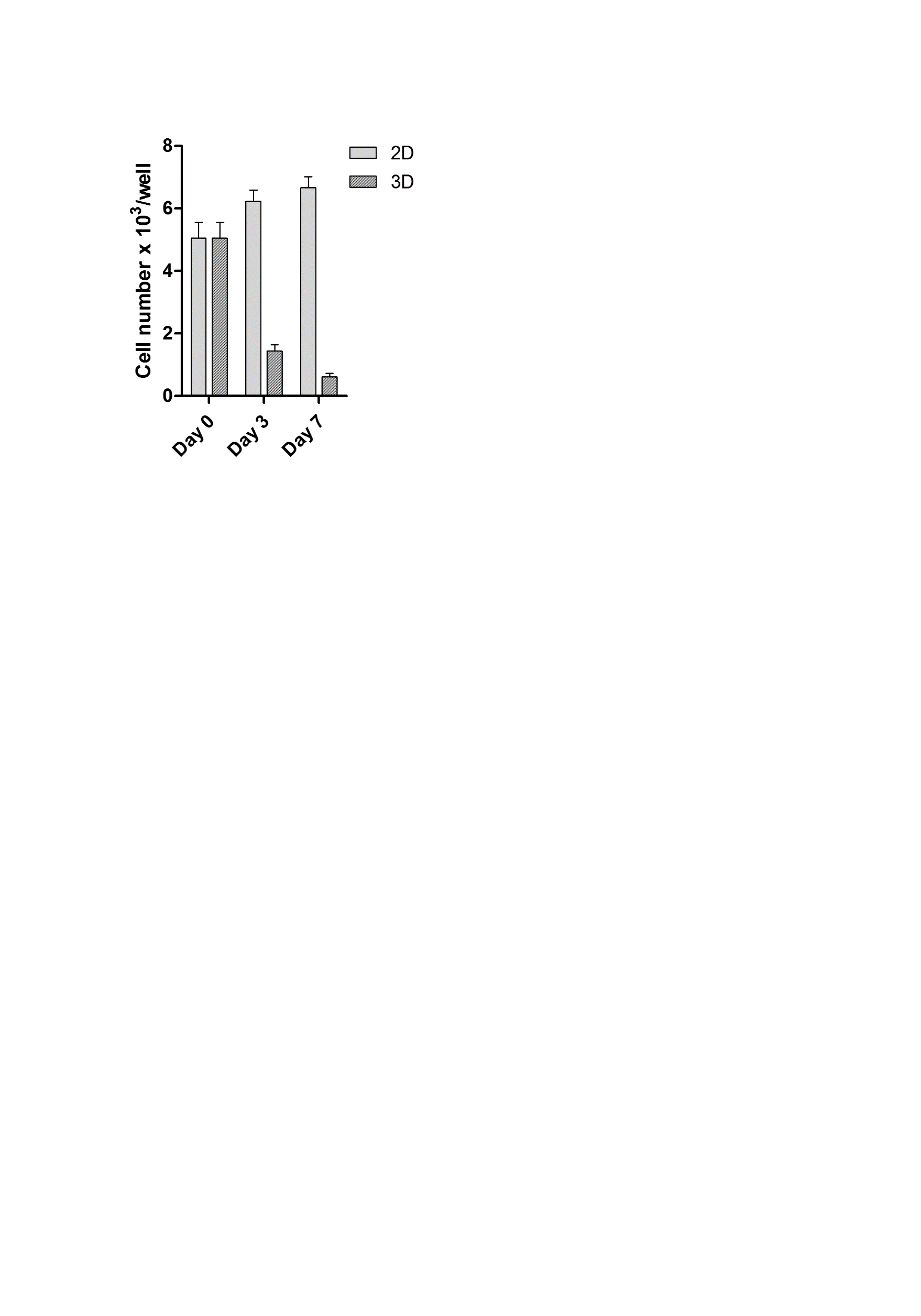


**Suppl. Fig. S1. MSC proliferation in GMP medium under 2D and 3D conditions.**

MSC growth as spheroids in hanging drop plates was compared to their culture as monolayers attached to the culture dishes: under both conditions MSCs were cultured in GMP medium conventionally used for MSC expansion. Cell numbers were determined by quantification of the DNA content. Data are shown as means ± SD of triplicate analysis and are representative of three donors with comparable results. MSCs continued to proliferate under 2D conditions, whereas considerably reduced cell numbers were detected in 3D spheroids after 3 and 7 days.


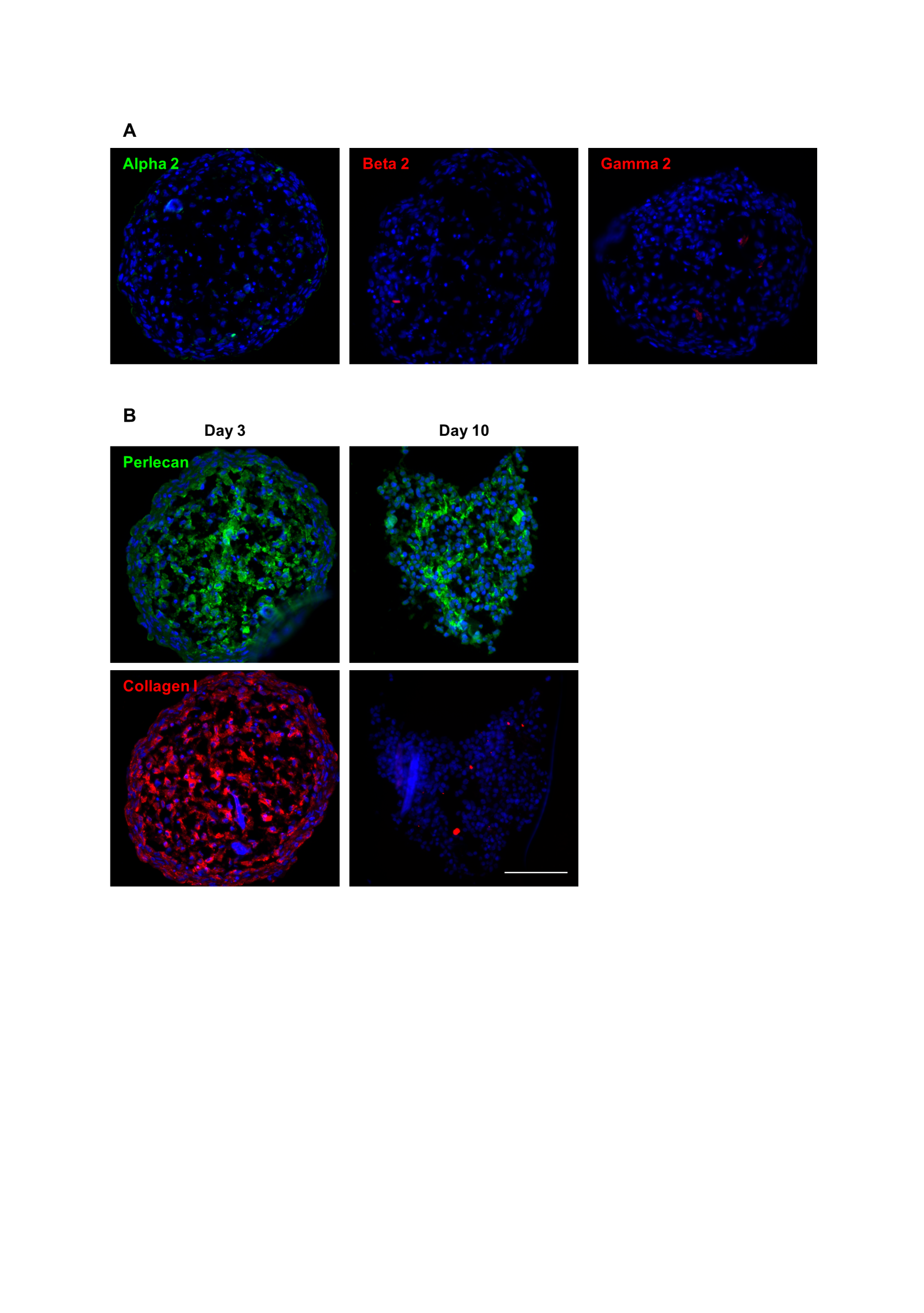


**Suppl. Fig. S2. Spheroid-forming MSCs synthesize ECM components of bone marrow.**

(A) Cryostat sections of MSC spheroids after 3 days in culture were labeled with chain-specific antibodies recognizing the laminin alpha2, beta2, and gamma2 chain. The laminin alpha2, beta2 and gamma2 chains could not be detected. (B) In contrast, high amounts of the basement-membrane component perlecan and the bone-specific collagen type I were synthesized in early spheroids. After 10 days of co-culture, perlecan was still present in the aggregates whereas collagen type I was not detectable. Cell nuclei were counterstained with DAPI. Scale bar, 100 µm.


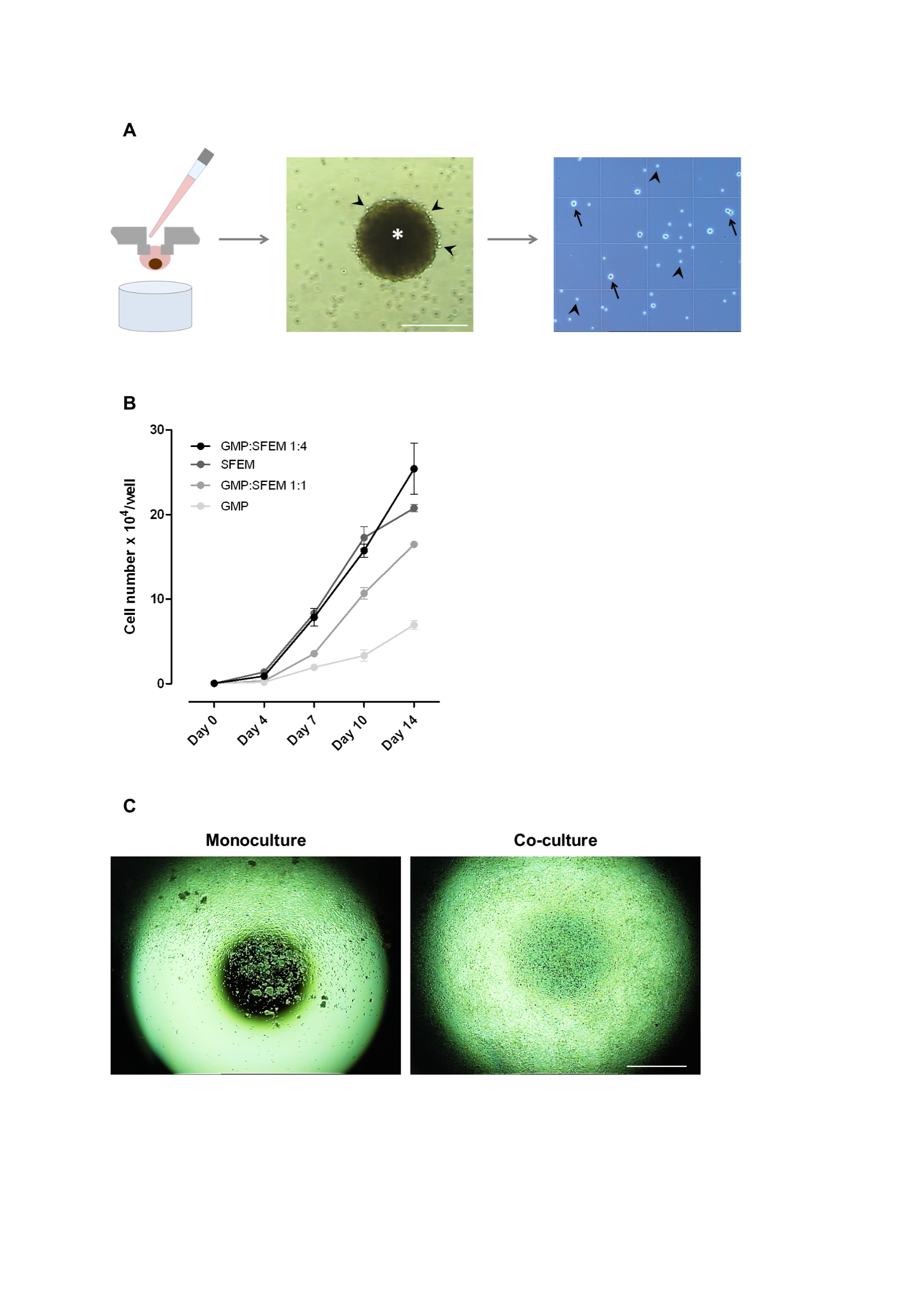


**Suppl. Fig. S3. Expansion of HSPCs in 3D hanging drops in comparison to 2D culture.**

(A) For the analysis of HSPC proliferation, cells from hanging drop plates were transferred to flat-bottom plates, which were placed below the hanging drop trays. High volumes of medium were pipetted from the top. The early MSC spheroids remained in their compact form (asterisk) with only a few attaching HSPCs (arrow heads) while the majority of HSPCs were in suspension, as seen using light microscopy. At later time points, aggregates consisting of apoptotic MSCs and invaded HSPCs could easily be separated into single cells by pipette mixing. HSPCs (arrow heads) were counted using a Neubauer chamber and were clearly distinguishable from MSCs (arrows) based on cell size, shape, and granularity. Scale bars, 250 µm. (B) In order to determine the optimal condition for HSPC expansion in hanging drops in co-culture with MSCs, different media compositions were tested. The highest cell number after two weeks of culture was obtained with a 1:4 mixture of GMP and SFEM supplemented with the CC100 cytokine cocktail. Data are mean ± standard deviation of three independent experiments. (C) By comparing the 2D and 3D cultures, a striking difference in the distribution of HSPCs grown in flat-bottom plates was observed: while cells in monoculture clustered to one side of the well, HSPCs incubated over a MSC monolayer were evenly distributed over the entire well area. Representative images were taken on day 14 of culture. Scale bar, 1 mm.
